# LINC00973 Induces Proliferation Arrest of Drug-Treated Cancer Cells by Preventing p21 Degradation

**DOI:** 10.3390/ijms21218322

**Published:** 2020-11-06

**Authors:** Dmitry S. Karpov, Pavel V. Spirin, Andrey O. Zheltukhin, Vera V. Tutyaeva, Olga L. Zinovieva, Evgenia N. Grineva, Vera A. Matrosova, George S. Krasnov, Anastasiya V. Snezhkina, Anna V. Kudryavtseva, Vladimir S. Prassolov, Tamara D. Mashkova, Nikolai A. Lisitsyn

**Affiliations:** 1 Engelhard Institute of Molecular Biology, Russian Academy of Sciences, 111991 Moscow, Russia; aleom@yandex.ru (D.S.K.); discipline82@mail.ru (P.V.S.); aozheltukhin@gmail.com (A.O.Z.); tutyaeva@gmail.com (V.V.T.); olezinovieva@mail.ru (O.L.Z.); grineva.ev@yandex.ru (E.N.G.); v.matrosova@gmail.com (V.A.M.); gskrasnov@mail.ru (G.S.K.); leftger@rambler.ru (A.V.S.); rhizamoeba@mail.ru (A.V.K.); prassolov45@mail.ru (V.S.P.); tamashkova@yandex.ru (T.D.M.); 2 Center for Precision Genome Editing and Genetic Technologies for Biomedicine, Engelhard Institute of Molecular Biology, 111991 Moscow, Russia

**Keywords:** LINC00973, DDR, CRC, 5-FU, p21

## Abstract

Overcoming drug resistance of cancer cells is the major challenge in molecular oncology. Here, we demonstrate that long non-coding RNA LINC00973 is up-regulated in normal and cancer cells of different origins upon treatment with different chemotherapeutics. Bioinformatics analysis shows that this is a consequence of DNA damage response pathway activation or mitotic arrest. Knockdown of *LINC0973* decreases p21 levels, activates cellular proliferation of cancer cells, and suppresses apoptosis of drug-treated cells. We have found that LINC00973 strongly increases p21 protein content, possibly by blocking its degradation. Besides, we have found that ectopic over-expression of LINC00973 inhibits formation of the pro-survival p53-Ser15-P isoform, which preserves chromosome integrity. These results might open a new approach to the development of more efficient anti-cancer drugs.

## 1. Introduction

Investigation of molecular mechanisms, which determine the resistance of cancer cells to anticancer drugs, holds promise for the development of more efficient chemotherapeutics that inhibit both tumor progression and relapse. Cancer cell resistance to chemotherapeutics (either pre-existing in part of tumor cells or acquired during drug treatment) is caused by numerous molecular mechanisms [1]. The short list includes blockade of drug penetration into the cell or activation of pumping out of the drug, as well as genetic and epigenetic changes that minimize the extent of target damage or constitutively activate drug-inhibited signaling pathways. Most of chemotherapeutics cause DNA damage in the S-phase. Generated DNA lesions are detected by ATM, ATR, and DNA-PKcs protein kinases, which activate the DNA damage repair pathway (DDR) that induces phosphorylation of nearly 700 protein targets [2]. This results in cell cycle and translational arrest followed by apoptosis of “unrepairable” cells. However, drug-resistant cancer cells treated with chemotherapeutics prevent cell cycle arrest, which is necessary for completion of DNA damage repair, and block apoptosis and autophagy [3,4]. Still, continuous drug treatment results in suppression of growth and proliferation of cancer cells due to up-regulation of *CDKN1A* encoded p21 inhibitor of cyclin-dependent kinases, which causes G1 arrest and cellular senescence [5].

Analysis of long non-coding RNAs, transcriptionally activated upon DNA damage, gave an additional impulse to the field [4,6]. Recently, we have discovered a significant increase in the expression of the long non-coding RNA LINC00973 in response to treatment of two colon cancer cell lines with three anticancer drugs both in vitro and in vivo [7]. In the current work, we have compared the phenotypes of HT-29 colon cancer cells with CRISPR-mediated LINC00973 knockdown (LINC00973 KD) and cells with ectopic LINC00973 over-expression (LV2). Obtained results demonstrated that LINC00973 is predominantly a nuclear RNA, which inhibits proliferation of normal and cancer cells by blocking S/G2 transition, inducing apoptosis in response to anti-cancer drugs.

## 2. Results

### 2.1. Bioinformatics Analysis of LINC00973 Expression in Cancer Cells Treated with Chemotherapeutics

In order to reveal LINC00973 possible functions, we have performed a large-scale bioinformatics search aimed at identifications of genes that correlate or anti-correlate with LINC00973. For this purpose, we have built an RNA-Seq database, which contains the results of treatment of eleven cancer cell lines of different origins and one normal diploid cell line treated with ten anticancer drugs. Table 1 shows the most pronounced changes in LINC00973 transcription (with |log_2_FC| > 1; mainly up-regulation). The list of the drugs that induce LINC00973 transcription includes DNA damaging agents (which cause formation of double stranded breaks or block topoisomerases 1 or 2), replication inhibitor 5-fluorouracil, and microtubule inhibitors that cause mitotic arrest [8]. Thereby, LINC00973 up-regulation occurs both upon activation of DNA damage response and at mitotic arrest.

Since p53 protein regulates both of these processes, we have compared LINC00973 transcriptional response to doxorubicin treatment in pairs of HCT-116 TP53 wild-type (WT) and TP53 knockout (KO) cells. Analysis of treated vs. untreated HCT-116 cells demonstrated nearly fourfold LINC00973 transcriptional up-regulation in TP53 WT, as compared to TP53 KO cells. This implies that LINC00973 transcription is probably p53-dependent. However, we have found no transcriptional changes of LINC00973 in pairs of HCT-116 cells treated with nutlin-3, which is an inhibitor of p53 degradation by E3 ubiquitin-ligase MDM2. Therefore, drug-induced transcriptional up-regulation of LINC00973 does not depend on total p53 quantity, but rather is a consequence of drug-induced p53 protein modifications.

Next, we have found strong co-expression of LINC00973 RNA with six mRNAs encoding major p53 targets in pairs of p53 WT and p53 KO CRC cell lines (HCT-116 and SW48) treated with doxorubicin (Table 2). We detected the strongest correlations in differential expression of LINC00973 and CDKN1A transcripts (Table S1). CDKN1A-encoded p21 protein plays a major role in cell cycle progression, cellular proliferation and senescence, as well as in DNA damage repair [15]. This implies that upon drug treatment, LINC00973 could participate in at least some of these p21-regulated processes. Besides, analysis of LINC00973 expression in colonic tumors [16] and in cancer cell lines established from breast tumors at various stages of cancer progression [17] demonstrated that LINC00973 over-expression predominantly occurs at late stages of tumor development.

Finally, we have analyzed RNA-Seq data obtained upon treatment of the H-STS cell line (derived from gastro-entero-pancreatic neuroendocrine tumor GEP-NET) with a panel of 107 low molecular weight chemical compounds inhibiting various signaling pathways [18]. This analysis demonstrated drastic over-expression of LINC00973 upon treatment of H-STS cancer cells with EGFR inhibitor afatinib (200-fold). Besides, we have found a 500-fold increase in LINC00973 transcription upon treatment of H-STS cells with alisertib, which is an inhibitor of the major EGFR pathway effector: Aurora kinase A (AURKA). These data imply a strong down-regulation of LINC00973 transcription in response to the EGFR pathway activation.

### 2.2. LINC00973 Subcellular Localization

**Figure 1:**
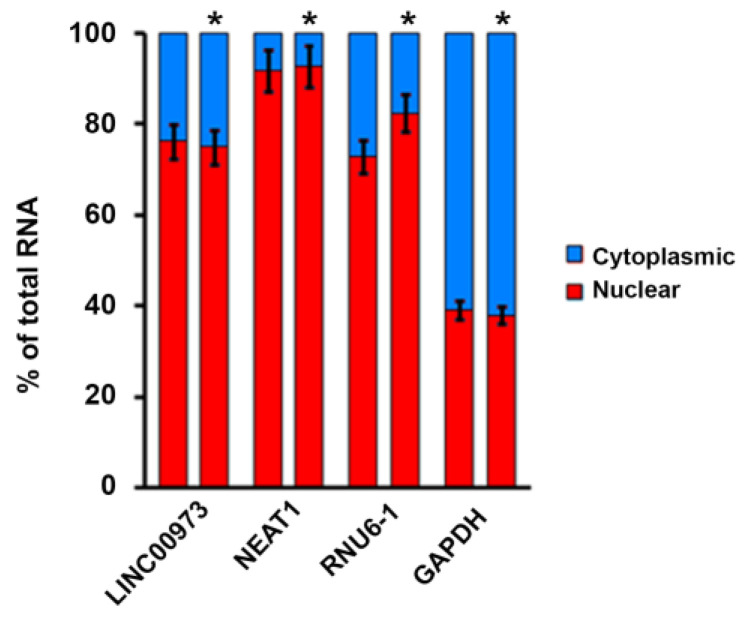
LINC00973 subcellular localization. The expression level of LINC00973 RNA was analyzed using RT-qPCR in total cellular RNA and subcellular RNA fractions of untreated and treated by 5-FU (*) HT-29 cells. NEAT1 and RNU6-1 lncRNAs (predominantly nuclear localization) and GAPDH mRNA were used as controls (gene-specific primers are shown in Table S2).

Since subcellular localization may provide a key to elucidation of lncRNA function, we have studied the LINC00973 RNA content in nuclear and cytoplasmic fractions employing fractionation test, both in 5-FU treated and untreated HT-29 cells. Obtained results demonstrated that LINC00973 RNA preferentially localizes to the nucleus (80%) and to a minor extent to the cytoplasm (20%) both in 5-FU treated and untreated cells (Figure 1). This implies that LINC00973 is most probably a nuclear scaffold RNA, which to a minor extent could function as a sponge or decoy in the cytoplasm.

### 2.3. LINC00973 Knockdown Results in Dramatic Increase in HT-29 Cell Proliferation and 5-Fluorouracil Sensitivity, As Well As in Inhibition of Apoptosis

To gain more detailed information about possible molecular functions of LINC00973, we sought to obtain cell lines with LINC00973 hypofunction. We have used CRISPR/Cas9 [19] in order to knockdown *LINC00973* gene by transfection of HT-29 subline (derived from a single HT-29 cell) with a plasmid that contained *Streptococcus pyogenes* Cas9 nuclease and dual guide RNAs for excision of coding sequence of the most abundant LINC00973 isoform (see Figure S1A). PXK cells (HT-29 cells transfected with an empty plasmid) were used as control. Using RT-qPCR, we have identified two clones (H1 and H2), in which we have found thirtyfold and sixtyfold drops in LINC00973 RNA content vs. PXK control, respectively.

**Figure 2:**
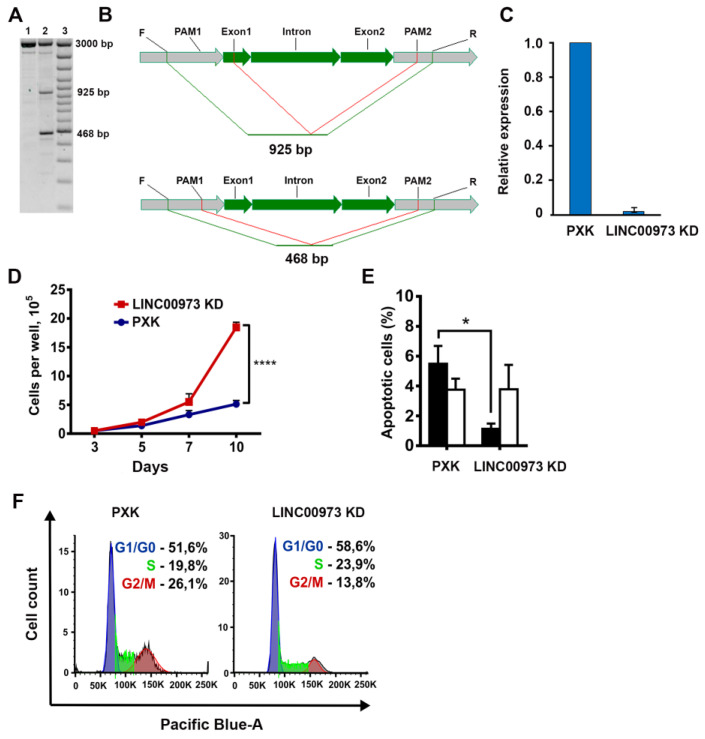
Analysis of the LINC00973 KD cell phenotypes. (**A**) Mapping of LINC00973 deletions in KD and control PXK cells by gel separation of PCR products of LINC00973 genomic sequence (primers—Table 3. Lane 1—PXK cells; lane 2—LINC00973 KD (above—the intact 3 kb long allele, below—two deletions of the remaining alleles); lane 3—DNA marker. (**B**) Scheme of the CRISPR-induced breaks in the *LINC00973* gene (shown in green). Flanking genomic regions are depicted in gray, red lines—flanking deletions, green lines—expected PCR products. (**C**) Relative expression of LINC00973 in KD vs. control PXK cells, as measured by RT-qPCR (mean ± SD). (**D**) Proliferation level of LINC00973 in KD vs. control PXK cells (**** *p* < 0.005). (**E**) Percentage of apoptotic cells in LINC00973 KD cell population. Black bars represent Annexin positive/PI negative cell population (early apoptosis); white bars—Annexin positive/PI positive cells at late stage of apoptosis (mostly dead cells). (**F**) Cell cycle distribution of LINC00973 KD and PXK cells (* *p* < 0.05).

PCR of LINC00973 encoding DNA sequence in clone H2 (further designated as LINC00973 KD) showed the excision of two out of three alleles of *LINC00973* gene in nearly triploid HT-29 cells (Figure 2A). In the first allele, gene excision removed the sequence between the two protospacer-adjacent motifs (PAMs, PCR amplicon length—468 bp) (Figure 2B). In the second allele, CRISPR deletion removed the sequence between the first exon and the second PAM (PCR amplicon length—925 bp). Sanger sequencing results were consistent with the observed gel mobility of PCR fragments. Thereby, the third allele of the LINC00973 in KD cells is probably transcriptionally silent, due to heterochromatization that makes it inaccessible to CRISPR/Cas9 system (Figure 2C).

Analysis of the LINC00973 KD cell phenotypes demonstrated twice more rapid proliferation, as compared to the control PXK cells (Figure 2D, *p* < 0.05). Next, we have found the fourfold drop in 5-FU IC50 of KD cells vs. control PXK cells (28 µM in PXK vs. 7 µM in KD cells). We have also detected twofold decrease in the quantity of LINC00973 KD cells in G2/M phase vs. control PXK cells (Figure 2F). Finally, we have found a fivefold decrease in the level of early apoptosis in the LINC00973 KD cells, as compared to the PXK control (Figure 2E). At the same time the quantity of mostly dead cells at late stages of apoptosis was almost identical in KD and control cells. Therefore, LINC00973 activates early apoptosis, blocks cellular proliferation, and enhances cell resistance to DNA damage. Besides, it retards cell cycle at G2/M phase, which results in time lag that is necessary for completion of the DNA damage repair.

### 2.4. Analysis of Transcriptomic and Proteomic Changes in LINC00973 KD and LINC00973 Ectopically Over-Expressing Cells

In order to obtain cells with high ectopic expression of LINC00973, we have transduced the HT-29 single cell subline with LINC00973- and GFP-containing lentivirus construct (Figure S1B). Transduced cells were sorted by FACS (cells transduced with an empty virus served as control). RT-qPCR demonstrated that expression of LINC00973 in selected clones is up-regulated 16 times vs. control. However, we have found no phenotypic differences between HT-29 cells ectopically over-expressing LINC00973 (LV2) and cells transduced with an empty lentivirus control (LV1). Thereby, we have performed RNA-Seq analysis of two pairs of transcriptomes: (1) LV1 vs. LV2 cells, as well as of (2) LINC00973 KD vs. control PXK cells (SRA BioProject PRJNA667313). Sequencing reads calculation demonstrated strong log_2_FC changes of four mRNAs in both pairs of transcriptomes, which were strongly associated with *LINC00973* expression: (1) CDKN2B and DCBL2 mRNA changes were positively associated with LINC00973 content; whereas (2) RNF157 and NEURL1B mRNA changes were opposite to the level of LINC00973 transcription (Table 3). A bioinformatics search demonstrated that these correlations can be also detected in normal and cancer cells of various origins treated by five anticancer drugs. This implies that LINC00973 is co-expressed with the first two genes and represses transcription of the second pair of genes. Importantly, changes in RNF157 mRNA and LINC00973 RNA content were always opposite even in LV2 cells, as compared to LV1 cells.

**Figure 3:**
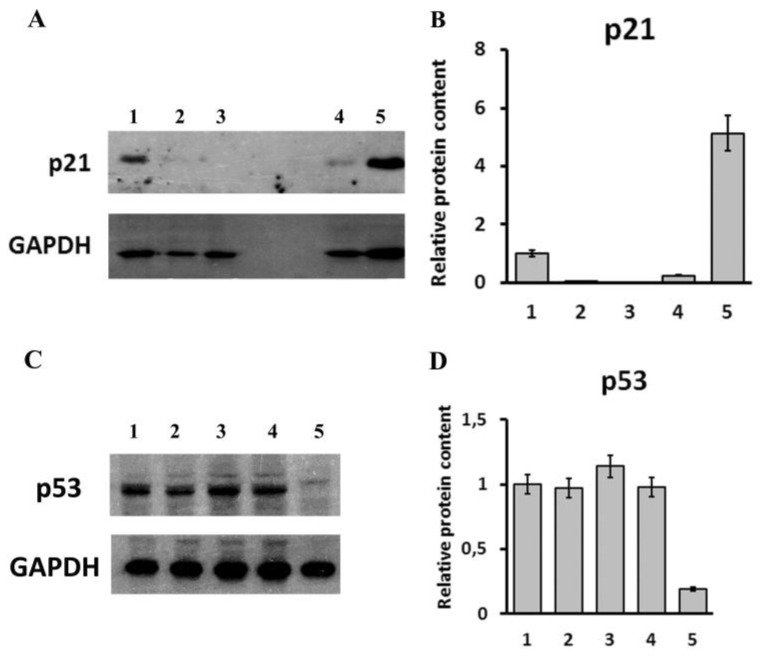
Top panel: Western blot (**A**) and densitometry analysis of p21 protein content. See the original in Figure S2. (**B**) in LINC00973 KD and LINC00973 ectopically over-expressing cells vs. controls: lane 1–PXK cells; 2 and 3—LINC00973 KD cells (clones H1 and H2, respectively); 4—HT-29 cells transduced with an empty lentivirus (LV1); 5—LINC00973 ectopically over-expressing HT-29 cells (LV2). Bottom panel: Western blot (**C**) and densitometry analysis of p53-Ser15-P protein content. See the original in Figure S2. (**D**) in LINC00973 KD and LINC00973 ectopically over-expressing cells vs. controls: lanes 1–5, as in top panel. GAPDH was used as an internal control.

Since p21 is nearly always co-expressed with LINC00973, we have analyzed p21 content in LINC00973 KD and LINC00973 ectopically over-expressing cells by Western blotting (Figure 3A,B). This experiment demonstrated that LINC00973 strongly up-regulates p21 content in absence of any significant changes in CDKN1A transcription (as follows from RNA-Seq data). This result implies that, upon drug treatment, LINC00973 RNA might retard p21-regulated cellular proliferation, providing additional time for DNA damage repair.

Finally, since p21 is a p53 target, we compared the level of the major p53 modification form that is induced in response to DNA damage (p53-Ser15-P) in LINC00973 KD and LINC00973 ectopically over-expressing cells vs. respective controls. The obtained results demonstrated a strong decrease in p53-Ser15-P level in LINC00973 ectopically over-expressing cells (Figure 3C,D). These data show that LINC00973 blocks formation of the major drug-induced form of p53 that is activated by ATM or CHK1/2 [20]. Thereby, LINC00973 could down-regulate the activity of these proteins.

## 3. Discussion

We have found that LINC00973 RNA is up-regulated upon treatment of normal and cancer cells with various chemotherapeutics, which cause activation of the DNA damage response pathway or mitotic arrest. Thereby, this RNA belongs to NORADs (lncRNAs activated by DNA damage) [21]. The first identified RNA from this family regulates genomic stability by sequestering PUMILIO proteins and its inactivation results in aneuploidy. However, possible functions of LINC00973 at least in part consist of an increase in p21 protein content by blocking its degradation, as well as in activation of the nearby DCBLD2 gene *in cis*. Besides, we have found that ectopic over-expression of LINC00973 in HT-29 cells inhibits formation of the pro-survival p53-Ser15-P isoform [22], which binds to centrosomes, preserving their integrity upon DNA damage and preventing chromosome defects [23]. This result is counterintuitive to obvious tumor suppressor functions of LINC00973. Nevertheless, there is a possibility that some other p53 modifications (from 52 discovered at the moment) take part in mitotic surveillance, replacing p53-Ser15-P. Obviously, this suggestion needs to be experimentally verified.

Recently, Liu et al. reported that LINC00973 RNA over-expression up-regulates SIGLEC15 mRNA in clear-cell renal carcinoma cells by sponging miR-7109-3p [24]. It was suggested, that SIGLEC15 membrane protein is a ligand of as yet unidentified T-cell receptor, which suppresses antigen-specific immune response. Our results on LINC00973 intracellular localization do not contradict this possibility. However, bioinformatics analysis clearly demonstrates the absence of changes in SIGLEC15 mRNA content in major adenocarcinomas, either treated or untreated with chemotherapeutics. This implies that LINC00973 functioning in RCC cells employs an unconventional mechanism, which needs to be experimentally explored.

**Figure 4:**
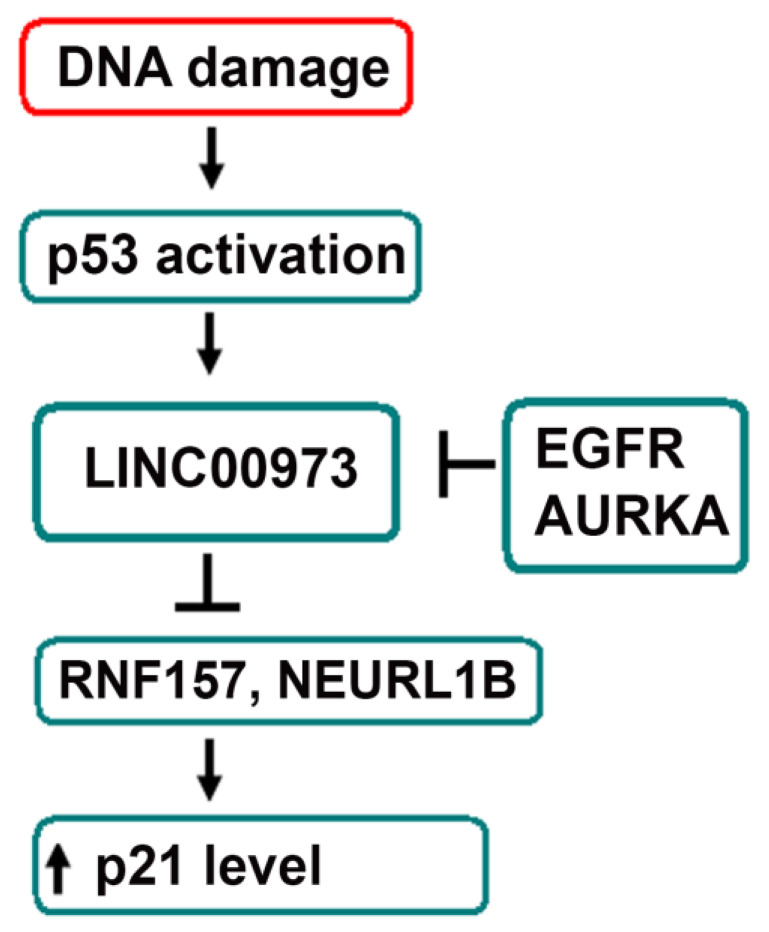
Hypothetical diagram of LINC00973 functioning. *LINC00973* expression is activated by p53 response to DNA damage. This results in LINC00973-mediated transcriptional repression of RNF157 and NEURL1B ubiquitin ligases, which leads to inhibition of p21 degradation.

In our opinion, LINC00973 exerts its anti-proliferative and pro-apoptotic functions in the following way (Figure 4). First, it is up to 100 times over-expressed in response to DNA damage. This might be a consequence of LINC00973 interaction with the activating transcriptional factor that, upon formation of a complex with the scaffold LINC00973 RNA, binds back to the *LINC00973* promoter, inducing over-expression of LINC00973 RNA. Second, LINC00973 RNA down-regulates transcription of *RNF157* and *NEURL1B* genes in co-operation with an as yet unidentified transcriptional repressor that pre-exists in a cell in absence of DNA damage (as follows from RNF157 transcription level comparison in LV2 vs. LV1 cells). Since both RNF157 and NEURL1B are E3 ubiquitin ligases, we speculate that they activate p21 degradation. Both of these genes are transcriptionally inhibited by LINC00973, which explains their anti-correlation with LINC00973 and strong p21 protein accumulation upon LINC00973 ectopic over-expression. MS analysis of RNF157 interacting proteins in melanoma cells [25] revealed its interaction with RNA binding protein MSI2 (Musashi RNA-binding protein 2), which is involved in cell cycle regulation and proliferation of cancer stem cells (CSCs). Therefore, LINC00973 might participate in down-regulation of CSC proliferation. These hypothetic conclusions need to be verified experimentally.

We have found that CDKN2B and DCBL2 mRNAs are co-expressed with LINC00973. This correlation could be explained by RUNX3 transcriptional regulation of both *LINC00973* and *CDKN2B* genes. Indeed, according to the UCSC ChIP-Seq database, the LINC00973 promoter and its three enhancers contain nearly a dozen strong RUNX3 binding sites, which were detected in several cell cancer lines. Thereby, co-expression of these two genes with LINC00973 could be explained by RUNX3-mediated transcriptional activation [26].

Besides, LINC00973 co-expression with DCBLD2 mRNA is hardly a coincidence. Both genes are neighbors on the third chromosome and LINC00973 is probably acting *in cis*, as an enhancer RNA or histone acetylase, which is necessary for *DCBLD2* transcriptional activation. Tenfold LINC00973 transcriptional up-regulation was observed upon treatment of colon cancer cells with EGFR-inhibiting antibody cetuximab [27]. These data are in accordance with the results demonstrating LINC00973 up-regulation upon treatment of GEP-NET cells with strong EGFR pathway inhibitors afatinib and alisertib. Absence of any mutations after the two-month cetuximab treatment of colon cancer cells implies that survived cells acquire resistance as a result of epigenetic changes activating LINC00973 transcription. However, the phenotypes observed upon inhibition of LINC00973 in CRC cells using siRNA in this report [27] (reduced proliferation/cell viability and increased apoptosis) were opposite to our observations. In our opinion, this is a consequence of much lower efficiency of siRNA vs. CRISPR knockdown technology.

Obviously, it is important to identify LINC00973 protein partners. This might provide new targets for development of the next generation chemotherapeutics that could activate LINC00973 effectors or to inhibit the RNF157 or NEURL1B protein activity that is suppressed by LINC00973. These compounds might significantly enhance the efficiency of conventional treatments, if used in combination.

In summary, we have shown that LINC00973 increases p21 protein content by blocking its degradation. This activates proliferation of cancer cells and suppresses apoptosis of drug-treated cells. These data might open a new approach in development of more efficient anti-cancer drugs, which eventually could overcome cancer cell drug resistance.

## 4. Materials and Methods

### 4.1. Analysis RNA-Seq Data and Data from the NCBI Sequence Read Archive

Reads were trimmed and filtered by trimmomatic 0.39 [28] and mapped to the reference human genome GRCh38 (Ensembl release 97) with STAR 2.7 [29]. Read counts per gene were estimated by the featureCounts tool from Subread package [30]. We evaluated 5′–3′ reads coverage distribution to ensure the absence of bias from RNA degradation, using geneBody_coverage script from RSeQC package [31]. EdgeR Bioconductor package [32] was used for differential expression analysis based on read counts. By default, we applied quasi-likelihood *F*-test with Benjamini–Hochberg *p*-value adjustment. Spearman’s rank correlation coefficients were estimated for various pairs of co-expressed genes.

### 4.2. Cell Lines and Drug Treatment

HT-29 human colorectal cancer cells and HEK293T cells (used for generation of lentiviral stock) were obtained from the Heinrich-Pette Institute, Leibniz Institute for Experimental Virology (Hamburg, Germany). Cells were cultured in Dulbecco’s modified Eagle’s medium plus 10% fetal calf serum, 100 units/mL penicillin, 100 μg/mL streptomycin and 1 mM sodium pyruvate at 37 °C and 5% CO_2_. HT-29 cells were treated with various concentrations of 5-FU (Sigma-Aldrich, Saint Louis, Missouri, USA) for 72 h.

### 4.3. RT-qPCR

Total RNA samples were extracted from cultured colon cancer cells using the RNeasy Mini kit (Qiagen, Limburg, The Netherlands). RNA integrity was evaluated by electrophoresis on 1% agarose gel and RNA quantity was determined using NanoDrop ND 1000 spectrophotometer (Thermo Fisher Scientific, Waltham, MA, USA). First-strand cDNA was synthesized using 1 µg of total RNA, random primers (Eurogen, Moscow, Russia) and SuperScript^TM^ III reverse transcriptase (Invitrogen, Carlsbad, CA, USA). Obtained cDNAs were amplified in the presence of gene specific primers, using the ABI 7500 Fast Real-Time PCR System (Applied Biosystems, Foster City, CA, USA). *ACTB* and *GAPDH* genes were used as controls. qPCR reactions were performed in triplicate in the presence of the EvaGreen^TM^ dye (Biotium Inc., Hayward, CA, USA) in the following conditions: denaturation at 95 °C for 10 min followed by 40 cycles of amplification (95 °C for 15 s, 60 °C for 1 min). Each plate included negative contamination control (in absence of cDNA); all experiments were repeated twice. Dissociation curve analysis was used in order to detect non-specific products. The relative expression ratios were calculated using the 2^−ΔΔ^*^Ct^* method [33].

### 4.4. LINC00973 Subcellular Localization

Total cellular RNA and subcellular RNA fractions were isolated from cells treated and untreated with 5-FU. Subcellular fractionation was performed with the SurePrep™ Nuclear or Cytoplasmic RNA Purification Kit (Fisher BioReagents, Canada) according to the manufacturer’s instructions. Cells grown in 100 mm plates were rinsed twice within ice-cold PBS and lysed in 200 μL of ice-cold lysis solution. After 5 min on ice, cell lysates were centrifuged at 12,000× *g* for 15 min at +4 °C. The supernatant was recovered in the cytoplasmic fraction, the pellet contained nuclear RNA. Purified total, cytoplasmic and nuclear RNAs were analyzed by RT-qPCR. Relative RNA abundance was corrected according to the portion of the total RNA present in each fraction. We have analyzed the expression of *LINC00973* and *DCBLD2* genes in total cellular RNA and subcellular RNA fractions. NEAT1, RNU6-1 (nuclear localization) and GAPDH (cytoplasmic localization) were used as controls.

### 4.5. LINC00973 Knockdown

Single cell HT29 clones were obtained by serial dilution in 96 well plates and LINC00973 mRNA content was measured by qRT-PCR. One of the clones with LINC00973 expression rate comparable to native HT29 cells was used for further experimentation. CRISPR/Cas9-mediated *LINC00973* deletion experiments were performed using pSpCas9(BB)-2A-Puro (PX459) V2.0 plasmid (a gift from Feng Zhang, Addgene #62988). Cells were transfected with either the plasmid encoding two sgRNAs for LINC00973 excision (PX459-anti-LINC00973, Figure S2), or with a control plasmid lacking sgRNAs. Transfection was performed in 6 cm dishes with Lipofectamine 2000 (Thermo Fisher Scientific, Waltham, MA, USA) according to the manufacturer’s protocol. Medium was changed 24 h after transfection to fresh Dulbecco’s modified Eagle’s medium (DMEM) containing 1 μg/mL puromycin and cells were incubated for 10 days. After serial dilution, single cell clones with LINC00973 knockdown cells were identified by qRT-PCR. Genomic deletions were mapped by Sanger sequencing, using PXK cells transfected with the plasmid lacking sgRNAs as control.

### 4.6. Viral Particle Production and Lentivirus Transduction

Viral stocks containing infectious pseudotyped viral particles were generated by transfection of 5 × 10^6^ HEK293T cells with: (1) 10 μg of lentiviral plasmid encoding LINC00973 (LV2) that was inserted into LEGO-iG2 vector or with an empty plasmid (LV1, used as a control). The transfection mix also included 10 μg of pMDL gag-pol plasmid, 5 μg of REV-encoding plasmid and 2 μg of pVSV-G expression vector. Cells were transfected using ProFection Mammalian Transfection System (Promega, Madison, WI, USA) and plated in a 10-cm Petri dish. Six hours after transfection, the medium was changed to DMEM containing 20 mM HEPES. After 16 h, supernatants containing viral particles were aspirated and filtered using Millex-GP 0.22 μM filter (Merk KGaA, Darmstadt, Germany). Titers were measured as described [34] and were found to be consistently higher than 5 × 10^6^ units/mL. Supernatants containing VSV-G pseudotyped viral particles and 8 μg/mL hexadimethrin bromide (Sigma-Aldrich, Saint Louis, MO, USA) were used to infect HT-29 cells at MOI = 0.15. Transduced HT-29 with ectopic over-expression of LINC00973 (LV2) and control cells expressing only GFP marker gene (LV1) were obtained by selection using BioRad S3 cell sorter (BIO-RAD, Hercules, CA, USA).

### 4.7. Analysis of Cellular Phenotypes

Analysis of LINC00973 KD, PXK, LV1 and LV2 cells was performed in the following way. To measure cell growth, cells were seeded into 24-well plates (10^4^ cells per well) and the amounts of cells in each well were counted with Neubauer chamber and Trypan Blue at days 3, 5, 7, and 10. Apoptosis was measured by double staining with Annexin V-Pacific Blue (Molecular Probes) and propidium iodide as described previously [35] on BD LSR Fortessa flow cytometer (Becton Dickinson, San Jose, CA, USA). FACS Diva v 5.0 software was used for data acquisition and post-acquisition data processing was carried out with FlowJo X software (Tree Star, Ashland, OR, USA). All reported values are means of three independent measurements, supplied with standard deviations.

To evaluate cell cycle distribution, 1 × 10^6^ of cells were harvested, washed with phosphate-buffered saline, and fixed with ice-cold 70% ethanol at −20 °C overnight. The next day, cells were washed with phosphate-buffered saline, incubated for 1 h in 100 μg/mL RNase A (Sigma-Aldrich, Saint Louis, MO, USA) and stained with 50 μg/mL propidium iodide (Sigma-Aldrich, Saint Louis, MO, USA). All measurements were performed using BD LSR Fortessa flow cytometer (Becton Dickinson, USA). Data analysis was performed as above.

### 4.8. Determination of IC50 upon 5-FU Treatment

Single cell subline of HT-29 cells and LINC00973 KD PXK cells were cultured in DMEM medium (Gibco, Waltham, MA, USA) with 10% FBS (Fetal Bovine Serum, BioSera, Rue de la Caille, France) at 37 °C and 5% CO_2_. Cells were passaged to avoid formation of the monolayer. Next, cells were seeded in the 96-well plate (1000 cells per well) and the next day they were treated by eleven twofold dilutions of 5-FU (Sigma-Aldrich, Saint Louis, MO, USA) (128 mkM–0.125 mkM). Medium was replaced daily in each of three days of treatment. Next, cells were washed with PBS and fresh medium was added, containing MTS reagent (Promega, Madison, WI, USA). After one-hour incubation absorption was measured at 490 nm at plate reader. MTS assay was performed three times and IC50 was measured using GraphPad Prism 6 software.

### 4.9. RNA Sequencing

Total RNA samples were isolated from HT-29 cells (control, transfected or transduced), using the MagNA Pure Compact Instrument (Roche Diagnostics, Indianapolis, IN, USA) according to the manufacturer’s instructions (the procedure included DNase treatment). Purified RNA samples were quantified with Qubit 2.0 Fluorometer (Thermo Fisher Scientific, Waltham, MA, USA) and sample quality was estimated by the calculation of RNA Integrity Number (RIN), using Agilent 2100 Bioanalyzer (Agilent Technologies, Santa Clara, CA, USA). RNA samples with a RIN higher than 8.0 were used for subsequent analysis. Poly (A^+^) mRNA fraction was isolated from 1 μg of total RNA samples using NEBNext poly(A) mRNA Magnetic Isolation Module (New England Biolabs, Ipswich, MA, USA). The cDNA library preparation was carried out using NEBNext Ultra Directional RNA Library Prep Kit and NEBNext Multiplex Oligos for Illumina (New England Biolabs, Ipswich, MA, USA) according to manufacturer’s instructions. The quality and concentration of cDNA libraries were assessed as described above; cluster densities were optimized by qPCR, using Rotor-Gene Q 5 plex platform (Qiagen, Limburg, The Netherlands). Obtained cDNA libraries were sequenced in triplicate on the Illumina NextSeq 500 platform under the 2 × 43 bp paired-end model, yielding 170 M mapped reads per experiment.

### 4.10. Identification and Ranking of Differentially Expressed Transcripts

This was completed as described before [22]. Briefly, Illumina reads were trimmed by trimmomatic [28], bacterial DNA/RNA contamination analysis was performed by mapping the sequencing reads to human rRNA and bacterial genomes databases using bowtie2. Obtained reports were summarized using MultiQC [36] and trimmed reads were aligned to human genome GRCh38 (Ensemble annotation, release 88) using STAR aligner [29]. Read counts per gene were estimated using HTSeq-count [37]. All processing steps were performed using PPLine pipeline [38] and the subsequent analysis was performed in R environment. Differential expression analysis was carried out using edgeR Bioconductor package [32]. Analysis of RNA-Seq data from the public NCBI SRA datasets (PRJNA417314, PRJNA315294, PRJNA355381, PRJNA335388, PRJNA392914, PRJNA421002, and PRJNA387040) was performed in the same way.

### 4.11. Western Blotting

Western blots were performed according to conventional protocols using p21 Waf1/Cip1 Rabbit mAb and Phospho-p53 (Ser15) Antibody (Cell Signaling, Beverly, MA, USA), GAPDH XP^®^ Rabbit mAb (Cell Signaling, Beverly, MA, USA) and peroxidase AffiniPure Goat Anti-Rabbit IgG (Jackson ImmunoResearch, Cambridgeshire, United Kingdom) as a secondary antibody. Amersham™ ECL Select™ Western blotting detection reagent (GE Healthcare, Chicago, IL, USA) was used for band visualization.

## Figures and Tables

**Table 1:** LINC00973 expression changes in response to chemotherapeutics.

Cell Line	Cell Type ^1^	Drug	Log_2_FC	P53	Reference
				Status	
HT-29	COAD	5-Fluorouracil	7.2	R273H	[7]
HT-29	COAD	Oxaliplatin	2.7	R273H	[7]
HCT-116	COAD	Oxaliplatin	2.8	wt	[7]
HCT-116	COAD	Doxorubicin	1.7	wt	[9]
SK-N-SH	NB	Doxorubicin	5.3	wt	[10]
SK-N-SH	NB	Cisplatin	5.2	wt	[10]
IMR5/75	NB	Doxorubicin	4.4	wt	[3]
U2OS	OS	Methyl methanesulfonate	−2.7	wt	[11]
U2OS	OS	Etoposide	−1.5	wt	[11]
pC9	LUAD	Carboplatin	2.5	R248Q	[12]
A549	LUAD	Paclitaxel	1.1	wt	[13]
IMR90	ILF	Etoposide	5.4	wt	[14]

^1^ COAD—colon adenocarcinoma; NB—neuroblastoma; OS—osteosarcoma; LUAD—lung adenocarcinoma; ILF—immortalized fetal lung fibroblasts. Shades of red indicate the degree of increase in the level of expression, while shades of blue indicate the degree of decrease in the level of expression.

**Table 2:** Log_2_FCs of LINC00973 RNA and mRNAs of p53 targets in two CRC cell lines treated with doxorubicin.

Cell Line	HCT116		SW48	
p53 Status	WT	KO	WT	KO
LINC00973	1.73	−0.17	0.68	−0.1
CDKN1A	3.11	0.19	3.33	−0.47
GDF15	2.5	−0.95	4.02	−0.33
BTG2	2.41	0.34	2.26	−0.02
BBC3	1.32	−0.71	1.83	−0.61
PMAIP1	1.51	0.61	0.68	−0.08
GADD45	1.65	−0.16	1.67	−0.05

WT—wild-type cell lines, KO—knockout cell lines. Shades of red indicate the degree of increase in the level of expression, while shades of blue indicate the degree of decrease in the level of expression.

**Table 3:** Associations in content of LINC00973 and four mRNAs revealed by RNA-Seq of normal and cancer cells upon drug treatment (log_2_FC).

Cell Line	HT-29			SK-N-SH		IMR90	pC9
Cell Type	COAD			NB		ILF	LUAD
Drug			5-FU	CIS	DXR	ETO	CAR
	LINC00973	LV2					
	KD vs. PXK	vs. LV1					
LINC00973	−5.64	4.51	7.15	5.24	5.3	5.39	2.49
RNF157	2.39	−2.11	−2.86	−2.86	−2.38	−1.19	−0.43
NEURL1B	2.06	0.1	−4.05	−2.59	−2.52	−3.53	2.39
CDKN2B	−1.51	0.23	2.19	3.26	3.43	2.13	2.5
DCBLD2	−2.01	0.1	1.9	2.29	2.74	0.18	−0.49

5-FU—5-Fluorouracil, CIS—Cisplatin, DXR—Doxorubicin, ETO—Etoposide, CAR—Carboplatin. Shades of red indicate the degree of increase in the level of expression, while shades of blue indicate the degree of decrease in the level of expression.

## Supplementary Materials

Supplementary Materials can be found at https://www.mdpi.com/1422-0067/21/21/8322/s1. Figure S1. Constructs used for modulation of LINC00973 activity: a plasmid encoding CRISPR/Cas9 system with dual sgRNAs for gene excision (A) and lentivirus vector for ectopic expression (B). Figure S2. Western blot analysis of p21 content. Figure S3. Western blot analysis of p53-Ser15-P content. Table S1. LINC00973 and CDKN1A expression changes in cancer cells treated with chemotherapeutics (log2FCs). Table S2. Primer sequences for RT-PCR.

## Abbreviations

LincRNA	Long intergenic non-coding RNA
DDR	DNA damage response
5-FU	5-Fluorouracil
OXP	Oxaliplatin
CIS	Cisplatin
CAR	Carboplatin
CSC	Cancer stem cell
PAC	Paclitaxel
ETO	Etoposide
DXR	Doxorubicin
RT-qPCR	Reverse transcription quantitative PCR
CPM	Counts per million
FC	Fold change
FDR	False discovery rate
CRC	Colorectal cancer
COAD	Colon adenocarcinoma
LUAD	Lung adenocarcinoma
NB	Neuroblastoma
OS	Osteosarcoma
ILF	Immortalized fetal lung fibroblasts
GEP-NET	Gastro-entero-pancreatic neuroendocrine tumor
LINC00973 KD	CRISPR-induced LINC00973 knockdown in PXK/HT-29 cells
PXK	HT-29 subclone derived from a single HT-29 cell, transfected with an empty plasmid PX459
