# Supplementary material for: LINC00973 Induces Proliferation Arrest of Drug-Treated Cancer Cells by Preventing p21 Degradation
Source: Int J Mol Sci. 2020 Nov 6;21(21):8322. doi: 10.3390/ijms21218322 (PMC7664178; doi:10.3390/ijms21218322)
Supplement: Supplementary file 1 [file ijms-21-08322-s001.zip › Supplementary Data/Supplementary Captions_v2.docx]

**Supplementary Figure 1.** Constructs used for modulation of *LINC00973* activity: a plasmid encoding CRISPR/Cas9 system with dual sgRNAs for gene excision (**A**) and lentivirus vector for ectopic expression (**B)**. The plasmid of the CRISPR/Cas9 system is derived from the pSpCas9(BB)-2A-Puro (PX459) plasmid (Addgene # 48139). The lentiviral vector is derived from the LeGO series of vectors (<http://www.lentigo-vectors.de/vectors.htm>).

**Supplementary Figure 2.** Western blots images obtained with Abs to p21 (left panel) and p53-Ser15-P (right panel). Lane 1 – PXK cells; 2 and 3 — LINC00973 KD cells (clones H1 and H2, respectively); 4 — HT-29 cells transduced with an empty lentivirus (LV1); 5 — LINC00973 ectopically over-expressing HT-29 cells (LV2).

**Supplementary Table 1.** LINC00973 and CDKN1A expression changes in cancer cells treated with chemotherapeutics (Log_2_FCs). Shades of red indicate the degree of increase in the level of expression.

**Supplementary Table 2.** Primer sequences for RT-PCR and verification of LINC00973 deletion.
