# Supplementary material for: LINC00973 Induces Proliferation Arrest of Drug-Treated Cancer Cells by Preventing p21 Degradation
Source: Int J Mol Sci. 2020 Nov 6;21(21):8322. doi: 10.3390/ijms21218322 (PMC7664178; doi:10.3390/ijms21218322)
Supplement: Supplementary file 1 [file ijms-21-08322-s001.zip › Supplementary Data/Supplementary Tables_v2.docx]

**Тable S1.** LINC00973 and CDKN1A expression changes in cancer cells treated with chemotherapeutics (Log_2_FCs).

| **Cell Line** | **HT-29** | **HCT116** | **SK-N-SH** | **SK-N-SH** | **IMR5/75** | **pC9** | **A549** | **IMR90** |
| --- | --- | --- | --- | --- | --- | --- | --- | --- |
| **Drug** | **5-FU** | **DXR** | | **CIS** | **DXR** | **CAR** | **PAC** | **ETO** |
| LINC00973 | 7.2 | 1.7 | 5.3 | 5.2 | 4.4 | 2.5 | 1.1 | 5.4 |
| CDKN1A | 3.3 | 3.1 | 5 | 4.3 | 5.6 | 1.9 | 2.1 | 2.1 |

**Table S2.** Primer sequences for RT-PCR.

| **Gene** | **F/R** | **Primer sequence (5’- 3’)** | **PCR product, bp** |
| --- | --- | --- | --- |
| LINC00973 | F | TTGAAGGCTTCCTGGTCTGAG | 162 |
|  | R | AGGCTTACATTCCAGCTGTGT |  |
| DCBLD2 | F | CCCACAGACCTATCCCAACAG | 195 |
|  | R | ATTTGCAACCCCAGACCACA |  |
| NEAT1 | F | TCGGGTATGCTGTTGTGAAA | 95 |
|  | R | TGACGTAACAGAATTAGTTCTTACCA |  |
| RNU6-1 | F | CTCGCTTCGGCAGCACA | 94 |
|  | R | AACGCTTCACGAATTTGCGT |  |
| ACTB | F | CCTTCCTGGGCATGGAGTC | 112 |
|  | R | CCAGACAGCACTGTGTTGGC |  |
| GAPDH | F | GGAGTCAACGGATTTGGTC | 181 |
|  | R | TGGGTGGAATCATATTGGAACAT |  |
| LINC00973 KD | F | GACCCTGGGCACTCTTGTTT | wt:3035  del 1:925  del 2:468 |
|  | R | TGAAACTGGCTTTCCTCAACC |  |
