# Supplementary figures and images for: LINC00973 Induces Proliferation Arrest of Drug-Treated Cancer Cells by Preventing p21 Degradation
Source: Int J Mol Sci. 2020 Nov 6;21(21):8322. doi: 10.3390/ijms21218322 (PMC7664178; doi:10.3390/ijms21218322)

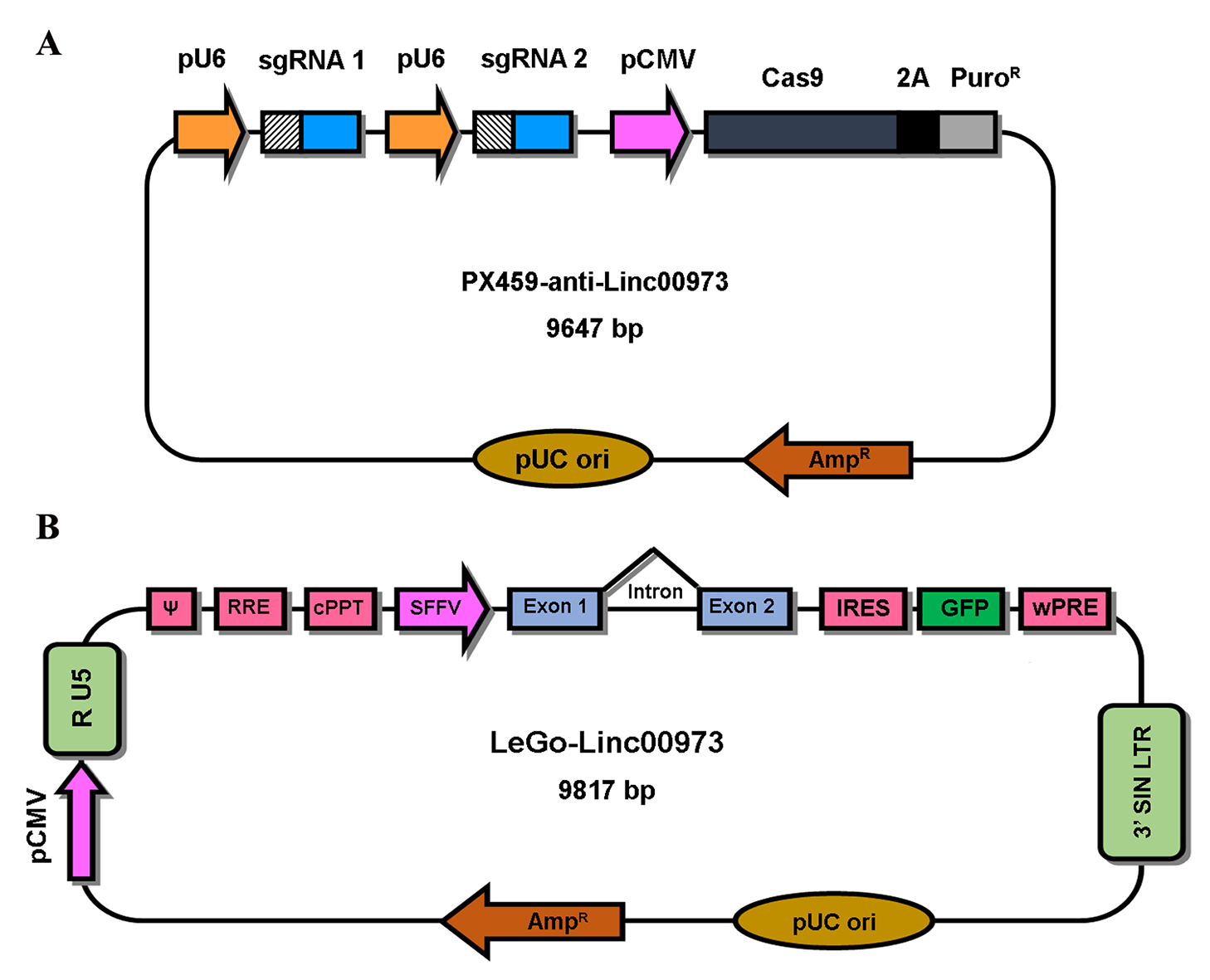

Supplement: Supplementary file 1 [file ijms-21-08322-s001.zip › Supplementary Data/Figure S1 Constructs_.tif]

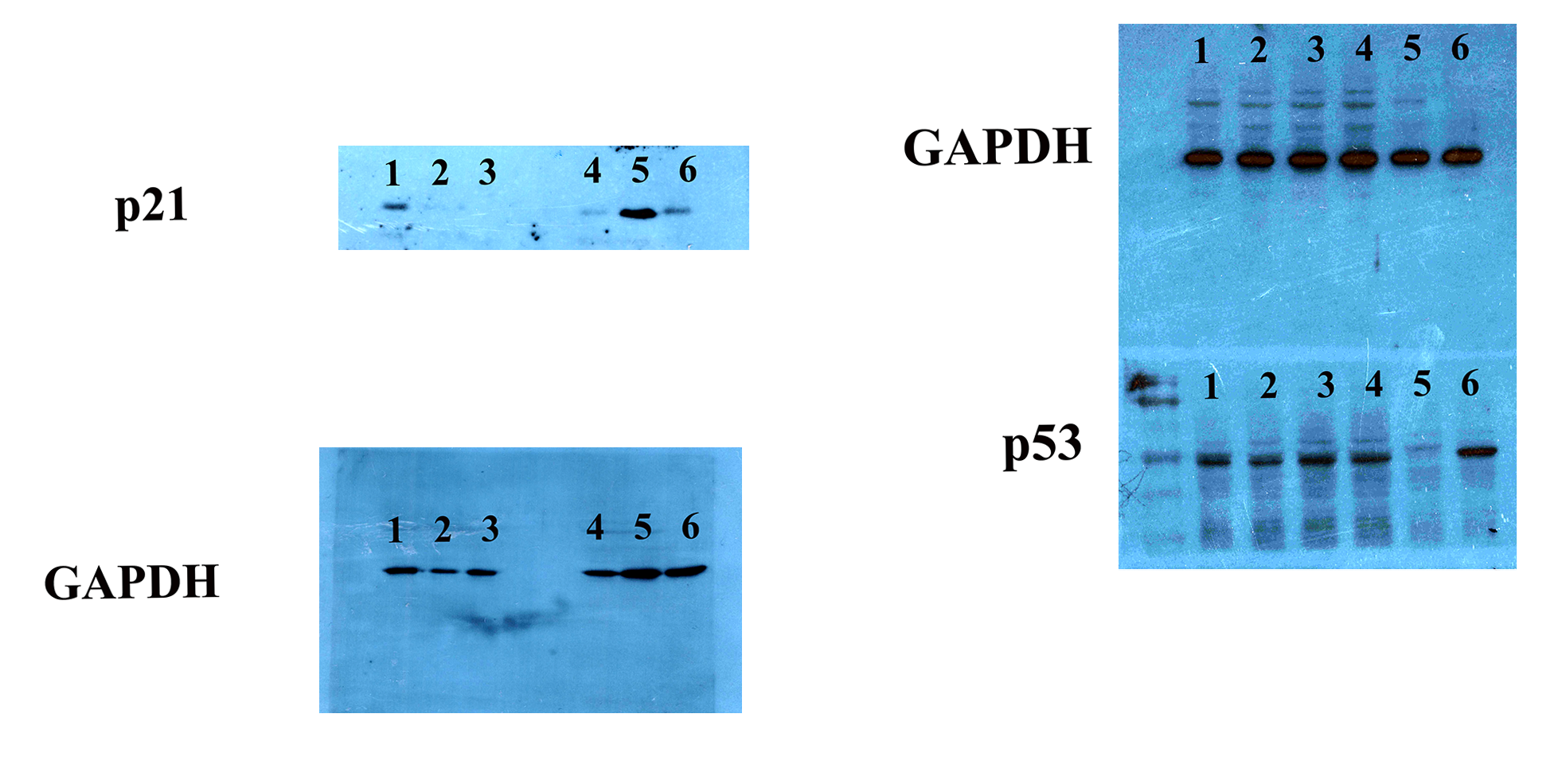

Supplement: Supplementary file 1 [file ijms-21-08322-s001.zip › Supplementary Data/Figure S2 Western blot_v3.tif]
